# Supplementary material for: Chemotactic behaviour of Escherichia coli at high cell density
Source: Nat Commun. 2019 Nov 25;10:5329. doi: 10.1038/s41467-019-13179-1 (PMC6877613; doi:10.1038/s41467-019-13179-1)
Supplement: Supplementary file 1 — Supplementary Information [file 41467_2019_13179_MOESM1_ESM.pdf]

Supplementary Information

Chemotactic behaviour of *Escherichia coli* at high cell density

Colin *et al.*

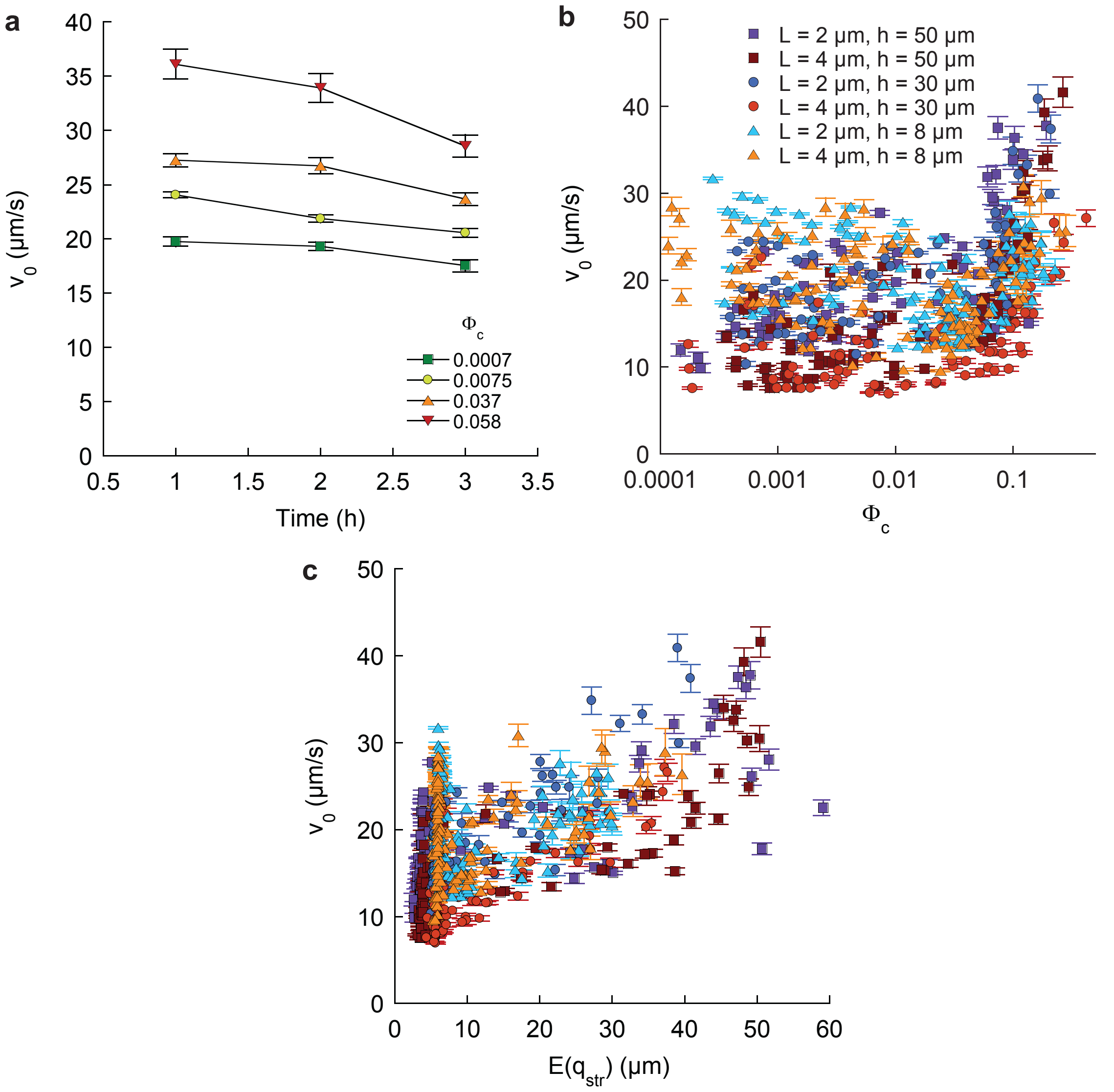

Supplementary Figure 1. (**a**) Examples of average swimming speed as a function of the time since sample preparation for normal cells in 50 μm high channels at indicated cell densities. The experiments were performed during the first three hours, for which swimming speed is fairly constant. (**b**) Average swimming speed, for all individual measurements, plotted as a function of cell body volume fraction. (**c**) The swimming speeds for all conditions were function of the amplitude of the collective motion, as defined in the main text, at high cell density. (**b-c**) Error bars represent measurement error.

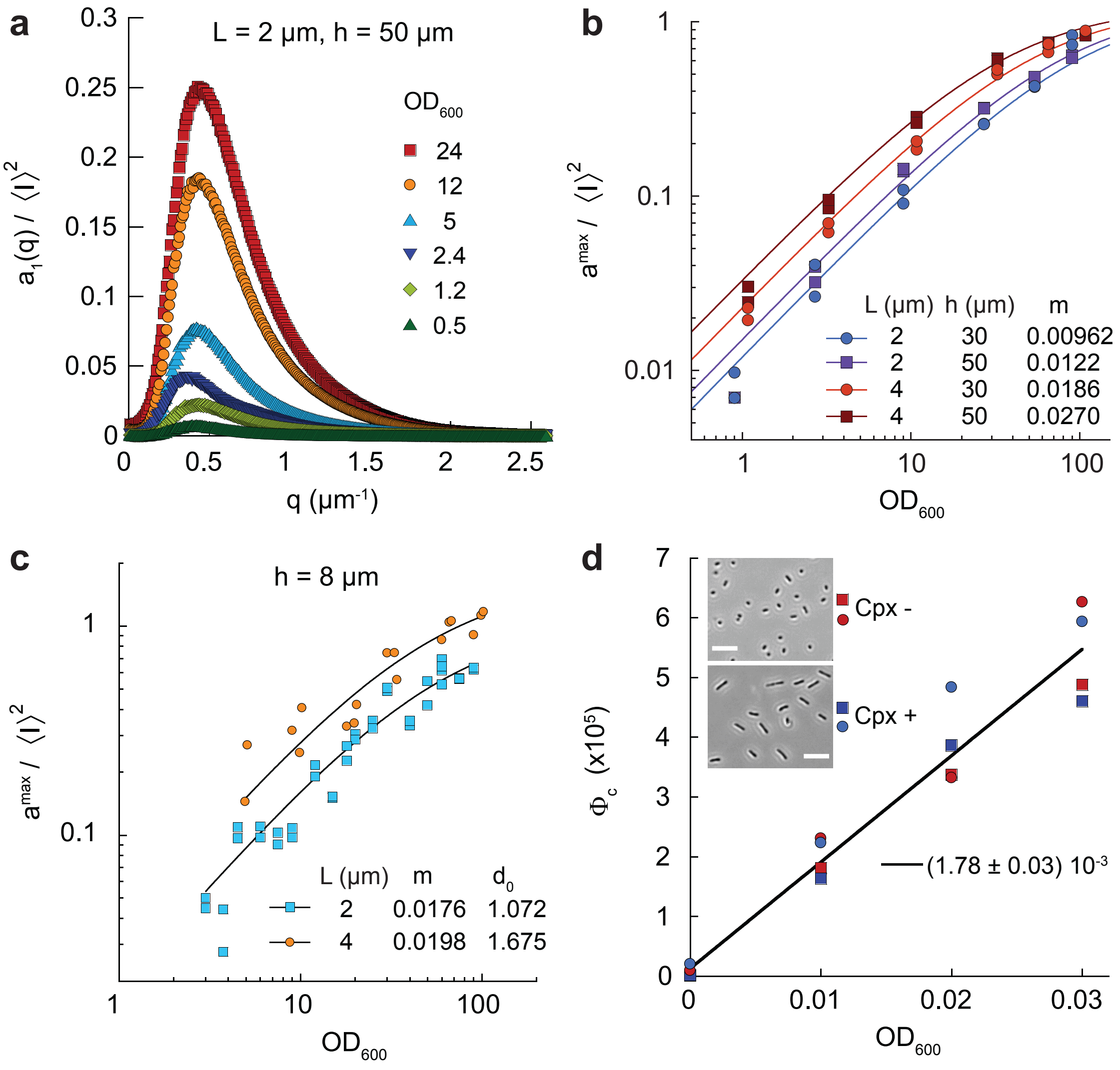

Supplementary Figure 2. *In situ* calibration of the cell body volume fraction. (**a**) The normalized amplitude of the differential intensity correlation function a_1/I^2, measured here in chemically homogeneous microfluidic devices, exhibits a maximum as a function of the spatial wave number q, which increases with the optical density. (**b**) The maximum value of a_1/I^2 can be fitted as $a_1^{\max}/I^2 = 1.25\, m\, \mathrm{OD}_{600}/(1 + m\, \mathrm{OD}_{600})$, with m depending on cell length L and channel height h, indicated in μm. (**c**) For channel height $h = 8\,\mu$m, the better adapted formula $a_1^{\max}/I^2 = d_0\, m\, \mathrm{OD}_{600}/(1 + m\, \mathrm{OD}_{600})$ was used. (**d**) The cell body volume fraction Φ_c was calibrated as a function of optical density for the single spectrophotometer used in the study, by cell counting in flow cytometry and length measurement in microscopy. The optical density was a single linear function of Φ_c, irrespective of cephalexin treatment. (Inset) Typical phase contrast images for cell length measurements, with and without cephalexin (Cpx) treatment. Scale bars are $10\,\mu$m.

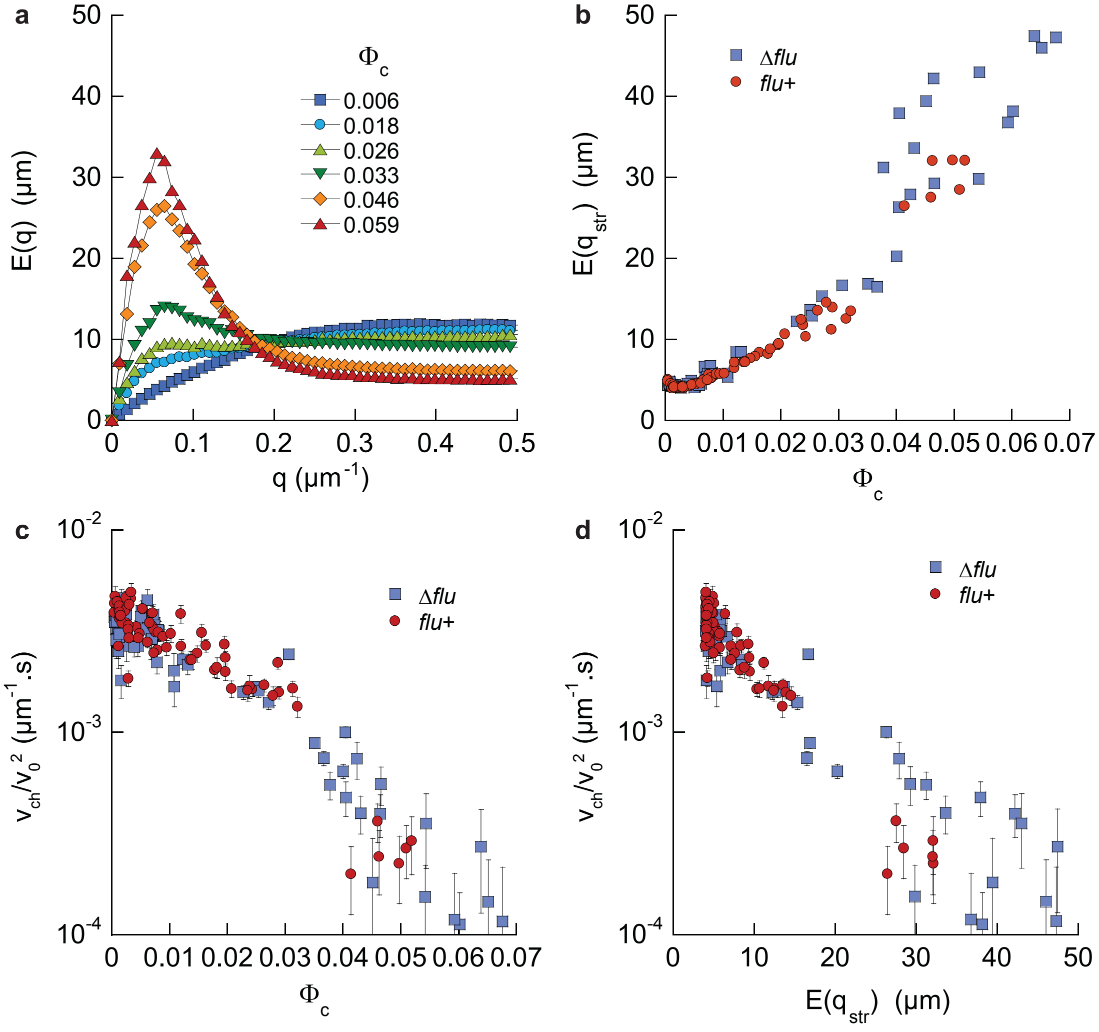

Supplementary Figure 3. Comparison of the behavior of $flu+$ and Δflu cells. (**a**) The spatial power spectral density E(q) measured in the 50 μm high channels exhibited a peak at $\pi/q_{str} = 50$ μm, as in the Δflu mutant, growing with cell density. (**b**) The maximum value of E(q) grew in exactly the same manner for both strains. (**c-d**) The normalized chemotactic drift had the same dependence on the cell body volume fraction (**c**) and on the amplitude of the collective motion E(q_{str}) (**d**) in both strains.

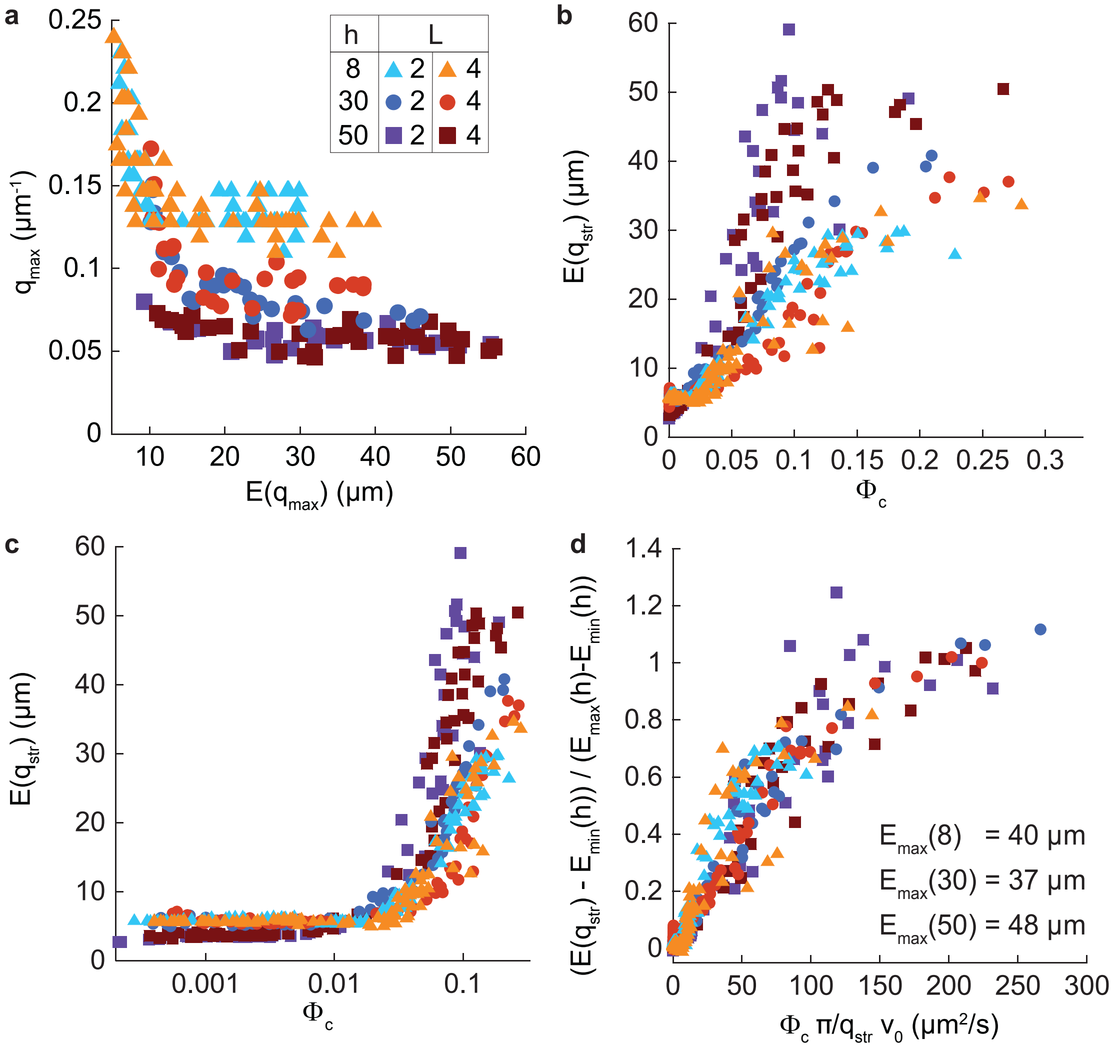

Supplementary Figure 4. Supplemental analysis of the collective motion. (**a**) Wave number q_{max} for which the maximum of E(q) is reached as a function of this maximum $E(q_{max})$, for all conditions. When the amplitude of the collective motion as quantified by $E(q_{max})$ is high, q_{max} reaches a plateau at q_{str}, which depends in first approximation only on the height h of the channel. Each point represents a single experiment. (**b**) Amplitude $E(q_{str})$ of the collective motion, measured at the plateau wave number $q_{str}(h)$, for each individual experiments, and used to draw Fig. 2**c**. We used $q_{str}(8\,\mu m) = 0.15\,\mu m^{-1}$, $q_{str}(30\,\mu m) = 0.093\,\mu m^{-1}$ and $q_{str}(50\,\mu m) = 0.055\,\mu m^{-1}$. (**c**) Same as **b** with Φ_c in logarithmic scale. (**d**) $E(q_{str})$, when corrected for its low density value $E_{\rm min}(q_{\rm str}(h))$ and normalized to the value at which it saturates at large density $E_{\rm max}(h)$, appears to be a single function of the cell body volume fraction times typical vortex size times swimming speed ($\Phi_c\,\pi/q_{\rm str}\,v_0$).

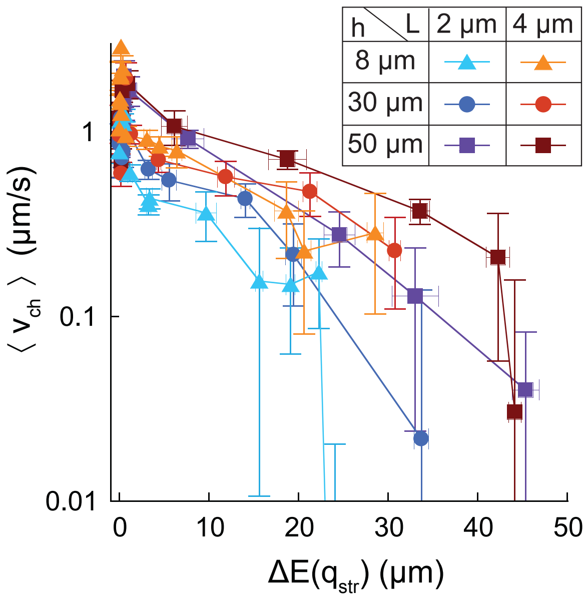

Supplementary Figure 5. Chemotactic drift $v_{\rm ch}$ as a function of the amplitude of the collective motion $\Delta E(q_{str})$. It decreases in all experimental conditions.

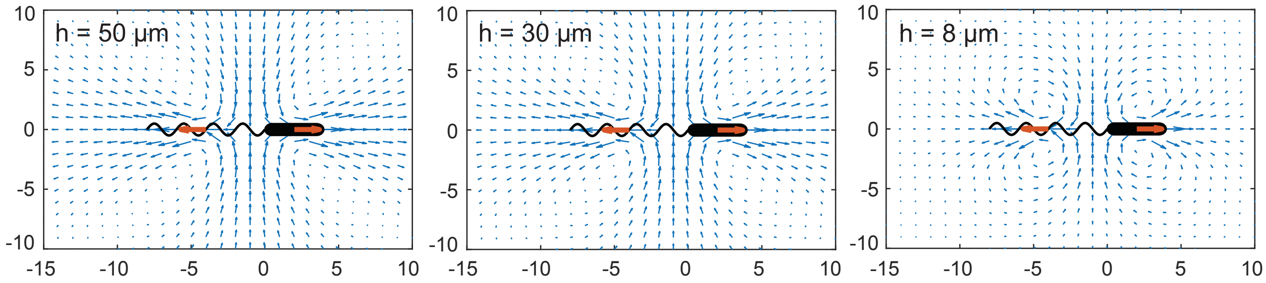

Supplementary Figure 6. Flow field generated around a swimmer for the three simulated heights.

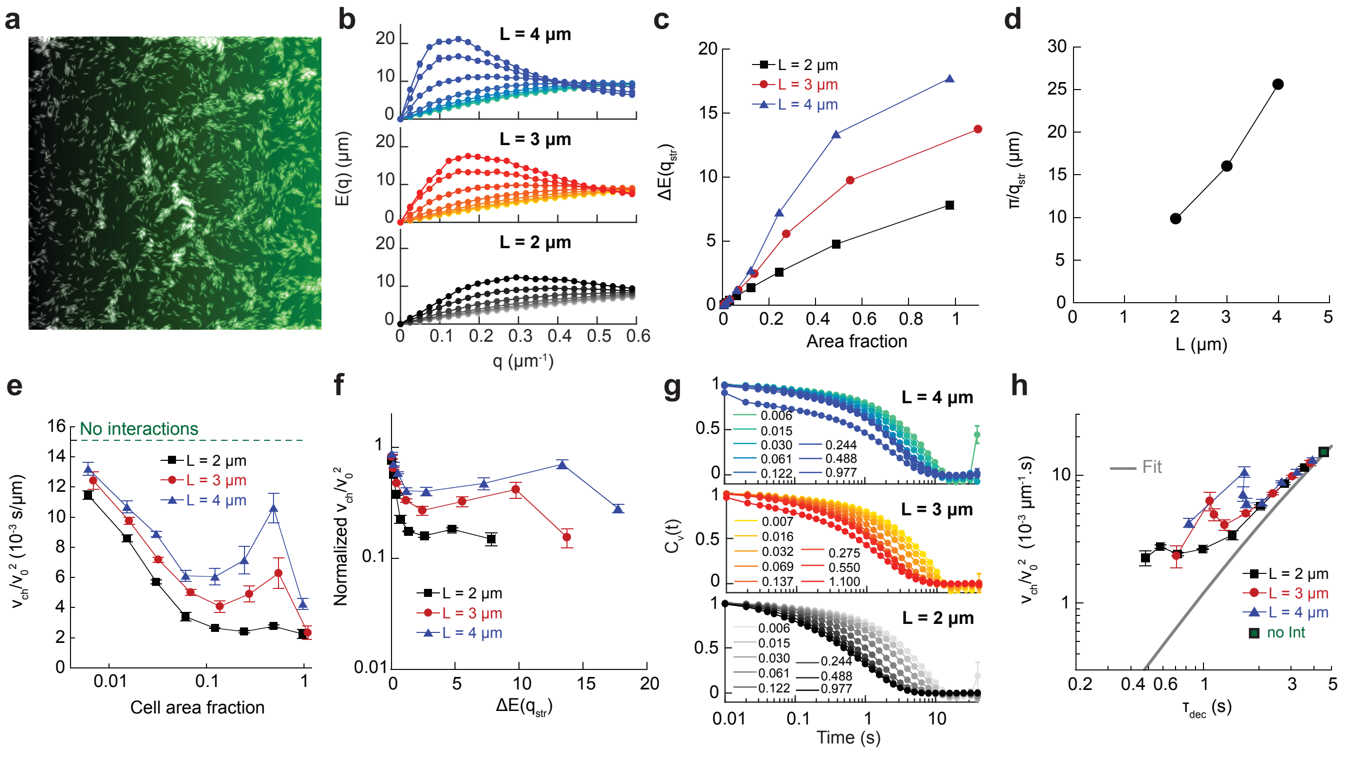

Supplementary Figure 7. Numerical simulations of self-propelled chemotactic rods considering only steric interactions. (**a**) Snapshot of a simulation, with linear gradient imprinted in green (area fraction 0.244, $L = 4$). (**b**) Flow structure factor $E(q)$ for increasing volume fractions and indicated aspect ratios, showing a peak at q_{str} scaling with the aspect ratio. (**c**) Maximum $E(q_{str})$, subtracted of its low density value, as a function of cell area fraction. Note that contrary to experiments and the wet case, $E(q_{str})$ grows more slowly for shorter cells. (**d**) The typical size of the vortices depends on the cell aspect ratio, contrary to experiments and the wet case. (**e**) Chemotactic coefficient as a function of cell area fraction for the indicated aspect ratios. The green dotted line represents the value in absence of interactions. As in the experiments, it decreases and exhibit a peak for large aspect ratios. (**f**) Chemotactic coefficient as a function of the amplitude $E(q_{str})$. (**g**) Time autocorrelation of the cell velocity $C_v(t)$ for the indicated cell area fractions and aspect ratios. (**h**) Chemotactic bias $v_{\rm ch}/v_0$ as a function of the normalized decorrelation time. The gray line represents the fit by Eq. 4 of the main text. The green dot represents the value in absence of all interactions.

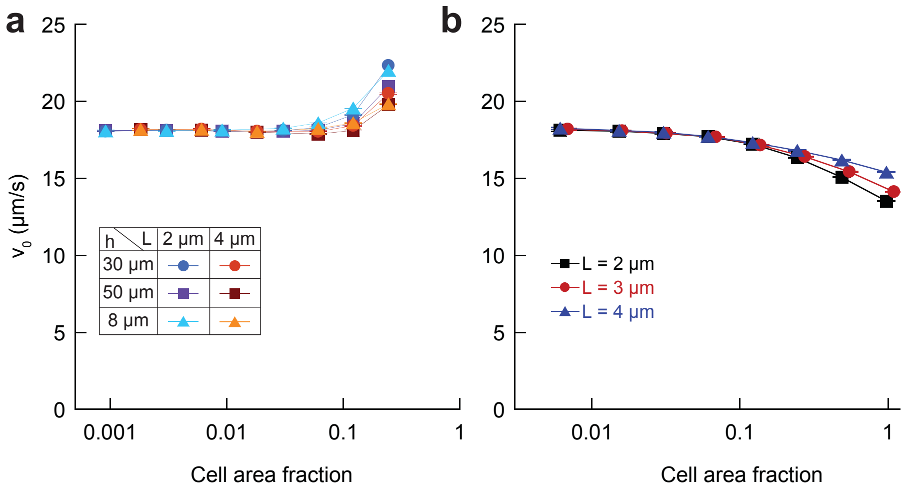

Supplementary Figure 8. Average swimming speed of the cells in the simulations in presence (**a**) or in absence of hydrodynamic interactions (**b**) as a function of cell volume fraction, for indicated cell lengths L, and channel heights h in the hydrodynamic case.

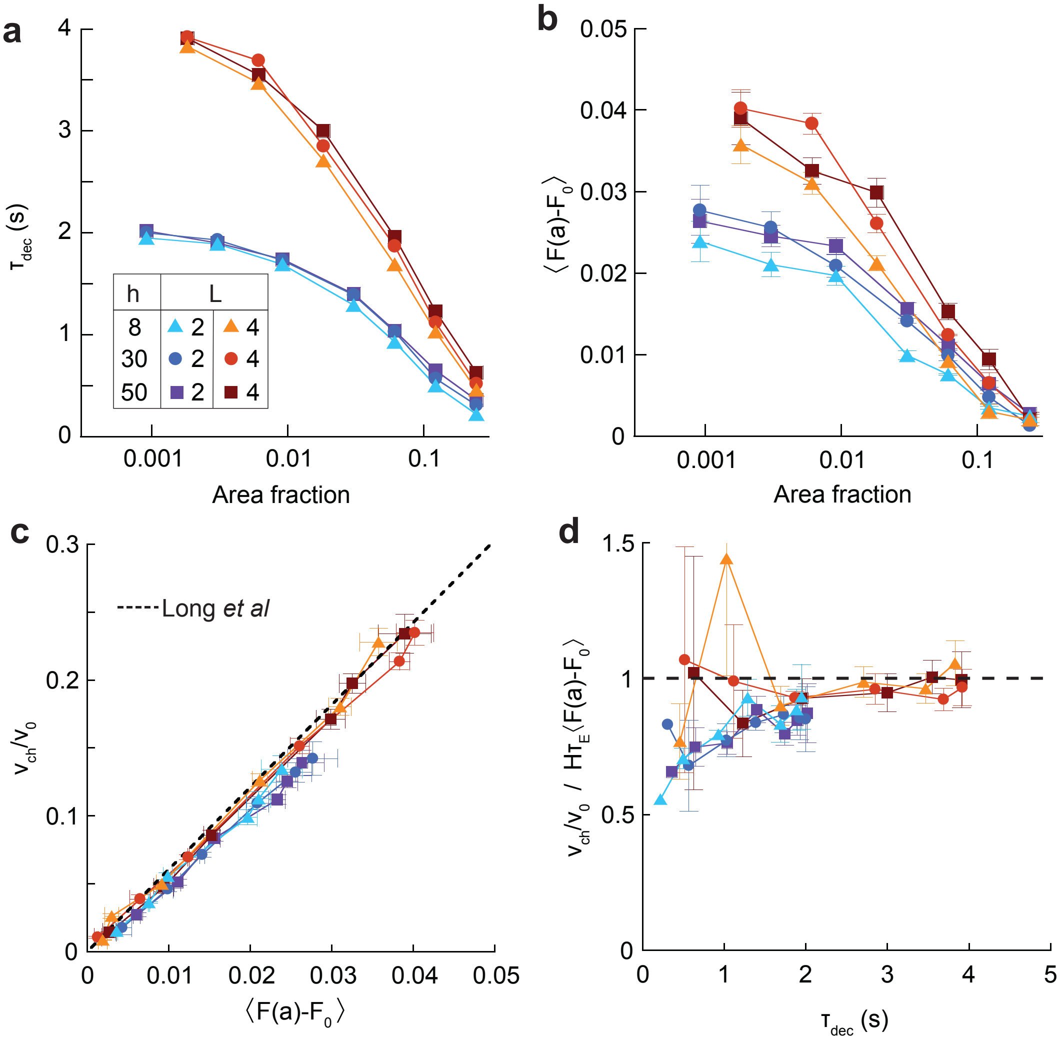

Supplementary Figure 9. Decorrelation time analysis in the simulations including hydrodynamics. (**a**) Decorrelation time, taken as $C_v(\tau_{\text{dec}}) = 0.5$ for the indicated simulation parameters. (**b**) Chemoreceptor free energy shift (CFES) as a function of area fraction (**c**) The chemotactic bias as a function of the CFES. The dotted line is the prediction of Long *et al.* [1]. (**d**) The normalized bias divided by the CFES as a function of the decorrelation time.

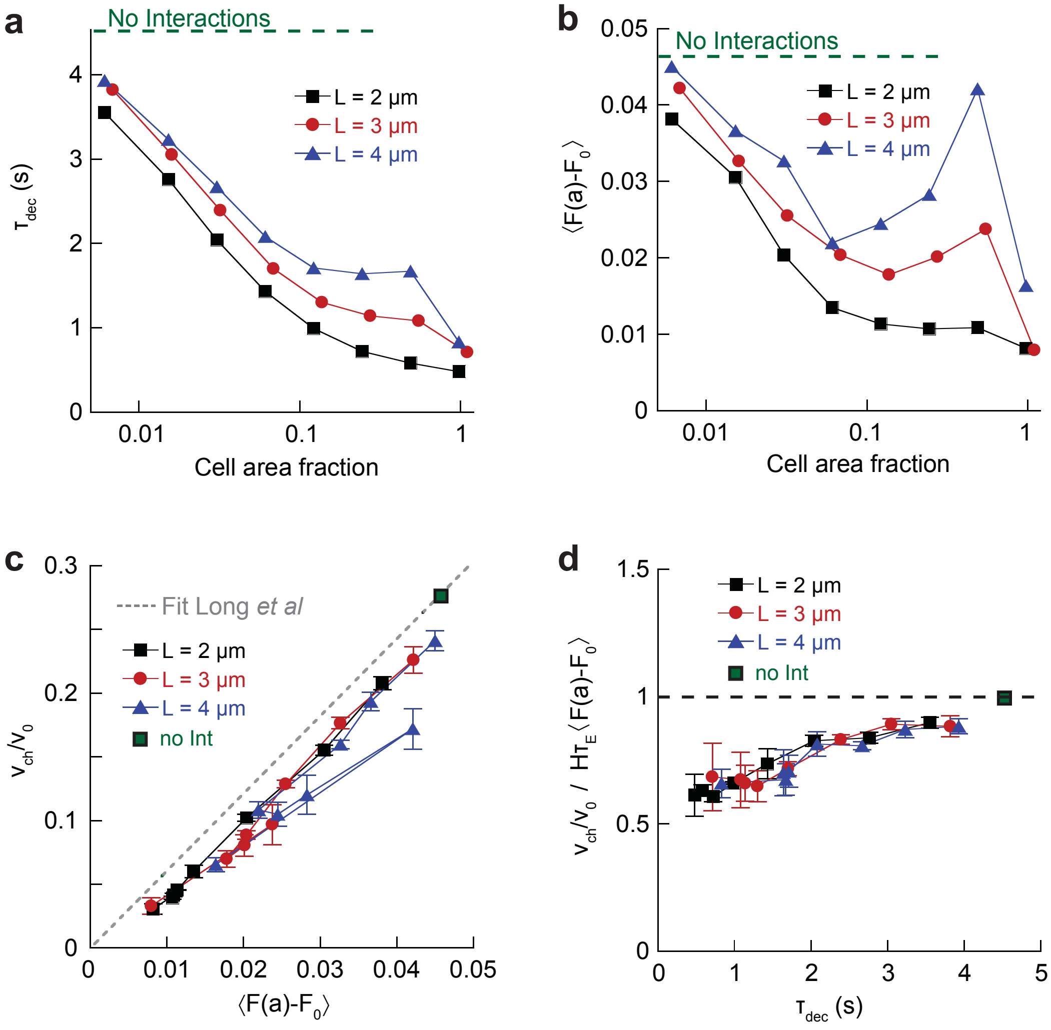

Supplementary Figure 10. Decorrelation time analysis in the dry simulations. (**a**) Cell velocity decorrelation time $\tau_{\rm dec}$, defined by $C_v(\tau_{dec}) = 0.5$ as a function of the area fraction. (**b**) The chemoreceptor free energy shift (CFES) is non monotonous as a function of area fraction. (**c**) Normalized chemotactic bias as a function of the normalized CFES. The gray dotted line represents the linear expectation from theory satisfying detailed balance [1]. (**d**) The normalized bias divided by the CFES, representing the fraction of the drift explainable assuming detailed balance is satisfied, as a function of the decorrelation time. (**a-d**) The green dotted line (**a-b**) or dot (**c,d**) represents the value in absence of all interactions.

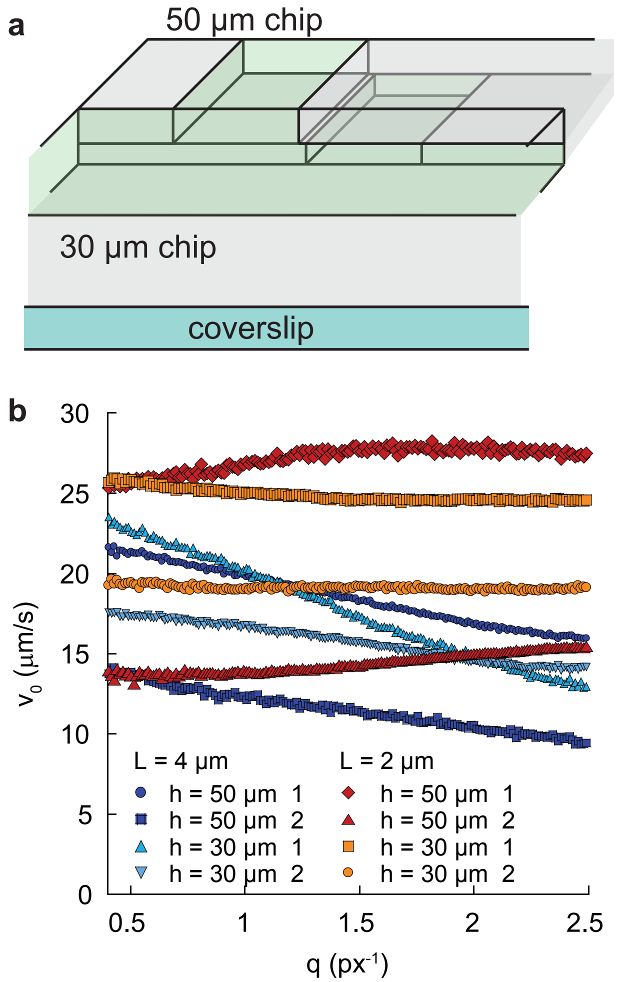

Supplementary Figure 11. Calibration of the velocity measurement by DDM. (**a**) Scheme of the geometry to compare DDM measurements of velocities in the 30 (or 8) μm and 50 μm devices. The two devices are facing each other and measurements can be done on the same suspension of cells in both channels. (**b**) Typical velocity outputs of the fit of the differential image correlation functions as a function of the wave number q, for the various experimental conditions. The effective velocity decreases for the longer cells, because of the anisotropy effect (see Supplementary note 5). The slight increase for the shorter cells was attributed to tumbles in earlier studies [2, 3].

Supplementary Table 1. Spincoating parameters

height (μm)	SU8	spincoat speed (rpm)
50	2050	1500
30	2050	3000
8	2015	2500

Supplementary Table 2. Parameters of the simulations

v_0	$20\,\mu$m.s^{-1}	
dv_0	$2\,\mu$m.s^{-1}	
δt	10^{-4} s	
1 frame	10^{-2} s	
$\nabla c/c_0$	$2\,10^{-4}\,\mu$m^{-1}	
c_0	$10^2\,\mu$M	
Chemotaxis pathway		
τ_r	1 s	
τ_t	0.1 s	
H	10	
N_a	10	
N_s	10	
$K_\mathrm{a}^\mathrm{off}$	$20\,\mu$M	
K_a^on	3 mM	
$K_\mathrm{s}^\mathrm{off}$	1 mM	
K_s^on	10 mM	
k_R	0.0182 s^{-1}	
k_B	0.0364 s^{-1}	
Z	31	
k_A	5	
k_Y	100	
Interaction parameters		
K_el/η	$10\,\mu$m$^{1/2}$.s^{-1}	
K_fr/η	$0.1\,\mu$m	
Cell parameters		
L	$2\,\mu$m	$4\,\mu$m
D_T	$6.7\,s^{-1}$	$4.2\,s^{-1}$
D_r	$0.1\,s^{-1}$	$0.0125\,s^{-1}$
L_flag	$10\,\mu$m	$10\,\mu$m
Modelisation parameters		
Gain G	$\tau_\mathrm{r}/(\tau_\mathrm{r}+\tau_\mathrm{t})\ HN_\mathrm{a}(1-P_\mathrm{on})/2$	
τ_m	$0.5(N_\mathrm{s}+N_\mathrm{a})P_\mathrm{on}(1-P_\mathrm{on})(k_\mathrm{R}+k_\mathrm{B})$	
$\nabla f(c)$	$\nabla c\left(1/(c_0+K_\mathrm{a}^\mathrm{off})-1/(c_0+K_\mathrm{a}^\mathrm{on})\right)$	
τ_0	$\tau_\mathrm{r}/(Y_\mathrm{p})^H$	
$1/H\tau_\mathrm{E}$	$N_\mathrm{a}\tau_\mathrm{m}v_0\nabla c/c_0$	

Supplementary notes

Supplementary Note 1. Peclet number

The Peclet number compares diffusion and advection of the chemical attractant in the fluid. It is defined as $Pe = hv_f/D$, since the typical size of the flow scales with the height of the channel. Here v_f is the velocity of the fluid. It can be estimated from the difference between the high density collective velocity and the free swimming speed assumed by the cell at low density. It never exceeded $10\,\mu$m/s (Supplementary Fig. 1). Taking the maximal $h = 50\,\mu$m, and considering that $D = 500\,\mu\text{m}^2$/s, leads to $Pe \leq 1$ for all experiments, no matter how strong the collective motion is. $Pe = 1$ indicates that distortions of the gradient are to be expected, as we observe in the case of the strongest collective motion.

Supplementary Note 2. Chemotactic drift in shallow gradients

Model of the pathway – The chemotaxis pathway of *E. coli* is composed of a large superstructure (array) of coupled receptor dimers, embedded in the inner membrane of the cell, and coassembled with the cytoplasmic kinase CheA and adaptor protein CheW. The most aboundant chemoreceptors of *E. coli* are Tar (the main sensor of MeAsp) and Tsr. Chemoeffectors bind the receptors on their periplasmic domain, inducing conformational changes which cooperatively modify the autophosphorylation activity of the kinase CheA, attractants reducing kinase activity. The cooperative array organization enables to integrate and amplify signals from different receptors. The kinase then transmits its phosphates to the small diffusible molecule CheY. Phosphorylated CheY binds to the motor to induce tumbles and is dephosphorylated by CheZ. The previous reactions are subsecond so that a sudden increase in attractant concentration induces a fast drop in the probability of tumbling. Two enzymes, CheR and CheB, then respectively add and remove methyl groups to specific amino-acids of the respectively inactive and active chemoreceptors in few seconds. This slowly offsets chemoeffectors action, adapting the average kinase activity – and thus the tumbling rate – back to the intermediate value it assumes in homogeneous environments, so that subsequent stimulations can be sensed. The current methylation level of the chemoreceptors then acts as a physical memory which represents the environment experienced by the cell a few seconds before, with which the current situation is compared.

We recall here the model of the chemotaxis pathway constructed in [4]. The chemoreceptor dimers were modeled as two-state variables interacting following a Monod-Wyman-Changeux allosteric model. The probability P_{on} of a signaling team of N_{a} Tar and N_{s} Tsr receptor dimers (and associated kinases) to be active is given by the free energy difference F between the active and inactive states as:

$$P_{\text{on}} = \frac{1}{1 + e^F} \tag{1}$$

with the free energy difference:

$$F = (N_{\text{a}} + N_{\text{s}})\,\epsilon(m) + N_{\text{a}} \ln\left(\frac{1 + c/K_{\text{a}}^{\text{off}}}{1 + c/K_{\text{a}}^{\text{on}}}\right) + N_{\text{s}} \ln\left(\frac{1 + c/K_{\text{s}}^{\text{off}}}{1 + c/K_{\text{s}}^{\text{on}}}\right), \tag{2}$$

where K_i^{off} (resp. K_i^{on}) is the binding affinity of the chemoattractant, present at concentration c, to the receptor i in its inactive (resp. active) state. The methylation dependent free energy difference $\epsilon(m)$ is

linear by part, and defined as:

$$\epsilon(m) = \begin{cases} 1.0 - 0.5\,m, & 0 < m < 2 \\ -0.3\,(m - 2.0), & 2 < m < 4 \\ -0.6 - 0.25\,(m - 4.0), & 4 < m < 6 \\ -1.1 - 0.9\,(m - 6.0), & 6 < m < 7 \\ -2.0 - (m - 7.0), & 7 < m < 8 \end{cases} . \tag{3}$$

The methylation enzyme CheR methylates only inactive receptors with average rate k_{R}, and CheB demethylates only active ones with average rate k_{B}, so that the methylation level evolves according to:

$$\frac{\mathrm{d}m}{\mathrm{d}t} = k_{\mathrm{R}}(1 - P_{\mathrm{on}}) - k_{\mathrm{B}} P_{\mathrm{on}} \tag{4}$$

The previous set of equations makes the fraction of active teams P_{on} evolve in time, responding and adapting to the history of concentrations experienced by the cell $c(t)$. The balance between autophosphorylation of active kinases and phosphotransfer to CheY sets the fraction of phosphorylated CheA dimers A_{p} as:

$$A_{\mathrm{p}} = \frac{k_{\mathrm{A}}\, P_{\mathrm{on}}}{k_{\mathrm{A}}\, P_{\mathrm{on}} + k_{\mathrm{Y}}} . \tag{5}$$

Finally, the fast phosphorylation – dephosphorylation cycle of CheY sets the fraction of phosphorylated CheY as:

$$Y_{\mathrm{p}} = 19.3610 \frac{k_{\mathrm{Y}}\, A_{\mathrm{p}}}{k_{\mathrm{Y}}\, A_{\mathrm{p}} + Z} . \tag{6}$$

If the cells are in the run state, the probability to tumble during the time step δt is given by:

$$p_{r \to t} = \exp\left(-\delta t\, (Y_{\mathrm{p}})^{H} / \tau_{\mathrm{r}}\right) \tag{7}$$

If the cell is in a tumbling state, the probability to start running again is however independent of the CheY phosphorylation level:

$$p_{t \to r} = \exp\left(-\delta t / \tau_{\mathrm{t}}\right) \tag{8}$$

All parameter values are given in Supplementary Table 2 and are as in [4] except for N_{a} and N_{s} which were chosen to match the values expected at the OD_{600} to which our cells are grown [5].

Prediction for the chemotactic drift in shallow gradients – We write here in our notations the results of Dufour *et al.* [6]. From Eq. 3 of this paper, because our simulations are 2D and the adapted value of Y_{p} is 1, we have:

$$v_{\mathrm{ch}} = (1 - TB) v_0^2 N_{\mathrm{a}} \nabla f(c)/2 \frac{\partial \ln \tau_{dec}}{\partial F} \frac{1}{1/\tau_{\mathrm{R}} + 1/\tau_{\mathrm{T}} + 1/\tau_{\mathrm{m}}} \tag{9}$$

were $(1 - TB) = \tau_{\mathrm{r}}/(\tau_{\mathrm{t}} + \tau_{\mathrm{r}})$ is the fraction of running cells at any given time in absence of a gradient, $\tau_{\mathrm{m}} = 0.5(N_{\mathrm{s}} + N_{\mathrm{a}}) P_{\mathrm{on}}(1 - P_{\mathrm{on}})(k_{\mathrm{R}} + k_{\mathrm{B}})$ is the relaxation time of F according to Eq. 2 and 4, $1/\tau_{\mathrm{dec}} = 1/\tau_{\mathrm{R}} + 1/\tau_{\mathrm{T}}$, and the derivative is taken at the adapted value $F = \ln 2$. We have also defined the Brownian reorientation time $\tau_{\mathrm{R}} = 1/D_{\mathrm{r}}$, and the tumbling reorientation time $\tau_{\mathrm{T}} = \tau_{\mathrm{r}}(Y_{\mathrm{p}})^{-H}/(1 - \exp(-D_{\mathrm{T}}\tau_{\mathrm{t}}))$. The

latter accounts for the incomplete randomization due to finite tumbling time [7], which was not accounted for in Dufour *et al.* [6] because complete randomization was assumed. We now considere that:

$$\frac{\partial \ln \tau_{\mathrm{dec}}}{\partial F} = -\tau_{\mathrm{dec}} \frac{\partial (Y_{\mathrm{p}})^H}{\partial F}(1 - \exp(-D_{\mathrm{T}}\tau_{\mathrm{t}}))/\tau_{\mathrm{r}} = -H \frac{\tau_{\mathrm{dec}}}{\tau_{\mathrm{T}}} \frac{1}{Y_{\mathrm{p}}} \frac{\partial Y_{\mathrm{p}}}{\partial F} \tag{10}$$

We also have from Eq. 1, 5 and 6 that:

$$\frac{1}{Y_{\mathrm{p}}} \frac{\partial Y_{\mathrm{p}}}{\partial F} = -(1 - \frac{k_{\mathrm{Y}} A_{\mathrm{p}}}{k_{\mathrm{Y}} A_{\mathrm{p}} + Z})(1 - \frac{k_{\mathrm{A}} P_{\mathrm{on}}}{k_{\mathrm{A}} P_{\mathrm{on}} + k_{\mathrm{Y}}})(1 - P_{\mathrm{on}}) \simeq -(1 - P_{\mathrm{on}}) \tag{11}$$

since the other factors are very close to 1, when one considers that they have to be evaluated for $F = \ln 2$. We then have:

$$v_{\mathrm{ch}} = v_0^2 (1 - TB) N_{\mathrm{a}} H (1 - P_{\mathrm{on}})/2 \frac{\tau_{\mathrm{R}}}{\tau_{\mathrm{R}} + \tau_{\mathrm{T}}} \frac{1}{1/\tau_{\mathrm{R}} + 1/\tau_{\mathrm{T}} + 1/\tau_{\mathrm{m}}} \nabla f(c) \tag{12}$$

which corresponds to Eq. 2 (and equivalently 4) of the main text, with the coefficients given in Supplementary Table 2.

Supplementary Note 3. Chemotactic behavior in simulations in absence of hydrodynamic interactions

Supplementary Fig. 7 shows the main characteristics of the collective motility and the drift in the simulations where hydrodynamic interactions are neglected. Note that the rotational Brownian motion was also neglected. The characteristics of the collective motion, as measured by the flow structure factor $E(q)$, were set by the cell length L (Supplementary Fig. 7**b-d**). The chemotactic drift first decreased with area fraction, before increasing, peaking and decreasing again for $L = 3\,\mu$m and $4\,\mu$m (Supplementary Fig. 7**e**). Contrary to the experiments, there was no scaling with $E(q_{\mathrm{str}})$ (Supplementary Fig. 7**f**).

Interestingly, the velocity decorrelation time τ_{dec} extracted from the time autocorrelation of the single cell velocity (Supplementary Fig. 7**g**) decreased monotonously in all conditions as a function of area fraction (Supplementary Fig. 10**a**), as it did when hydrodynamics was accounted for. However, in the dry case, the chemotactic coefficient, considering its dependence in τ_{dec}, did not follow Eq. 4 of the main text (Supplementary Fig. 7**h**), contrary to the full simulations (main Fig. 4**h**). Clearly, at the densities were the drift started to reincrease, the system started to depart from the framework of Eq. 4 of the main text, which assumes a Brownian motion like reorientation process and shallow gradients, i.e. that the average pathway activity P_{on} departs only slightly from its adapted value. Because it occurs at a fairly high cell density, it is however not clear if the peak observed here occurs for the same reasons as the one observed in the experiments.

Supplementary Note 4. Dual effect of physical interactions on the chemotactic drift

An extension of the model we used so far to explain chemotactic drift reduction (i.e. [6] leading to Eq. 2-4 of the main text) was proposed in Long *et al.* [1]. There, this extension was used to explain non-linear reinforcement of the chemotactic drift when the gradient is sharp enough. It therefore does not assume

shallow gradients anymore, and is based on a Schmolukovski equation for the space-averaged probability $P(t, F, s)$ to be at time t with a chemoreceptor free energy F and an orientation $s = \mathbf{n} \cdot \nabla c / ||\nabla c||$ [1]:

$$\partial_t P = -\partial_F \left(\left(-(F - F_0) + \frac{r(F)s}{H\tau_\mathrm{E}} \right) P \right) + \frac{\hat{L}_s P}{\tau_D(F)} \,. \tag{13}$$

Here time is normalized to the adaptation time τ_m and $r(F)$ is the probability of the cell being running, given the receptor free energy F. The first right hand side term describes the chemoreceptor free energy actuation according to adaptation (term $-(F - F_0)$ with F_0 the adapted, unstimulated, value of F; $F_0 = \ln 2$ in our case) and stimulation due to swimming in the gradient (with $1/H\tau_\mathrm{E}$ being the normalized gradient, defined in Supplementary Table 2 for our case). The second term describes reorientations due to Brownian rotational diffusion and tumbles, with $\hat{L}_s$ the rotational diffusion operator and $\tau_D = 1/\left(\tau_\mathrm{m}(rD_\mathrm{r} + (1-r)D_\mathrm{T})\right)$ comparing reorientation and adaptation times. Assuming that the detailed balance in the angular fluxes of cell orientation hold, we can include in this second term the effect of collective reorientations in the form of an effective enhanced rotational diffusion $D_\mathrm{r} \to D_\mathrm{r} + D_\mathrm{coll}^\mathrm{eff}$. Integrating this equation over orientations, in Long *et al.* [1] it is shown that at steady state in the gradient:

$$v_\mathrm{ch}/v_0 = \langle rs \rangle = H\tau_\mathrm{E} \langle F - F_0 \rangle \tag{14}$$

Here the chemotactic ratio v_ch/v_0 is the product of the normalized gradient strength and of the average over all positions, orientations and times of the shift in chemoreceptor free energy from its adapted value (CFES, $\langle F - F_0 \rangle$). Key assumptions for derivating this equation are steady-state and detailed balance holding. In this equation, the effect of reorientations (tumbles, Brownian motion and possibly collective reorientations) on the drift is accounted for by a reduction of the CFES. In the simulations (contrary to experiments), the CFES is readily accessible, and it evolves similarly to the drift as a function of volume fraction (Supplementary Figs. 9**b** and 10**b**). The chemotactic ratio v_ch/v_0 follows Eq. 14 as a function of $\langle F - F_0 \rangle$ fairly well in the simulations accounting for hydrodynamics (Supplementary Fig. 9**c**), but less so for the dry simulations (Supplementary Fig. 10**c**). Upon closer inspection using the ratio

$$\frac{v_\mathrm{ch}/v_0}{H\tau_\mathrm{E}\langle F - F_0 \rangle} \tag{15}$$

we find that the ratio decreases in both cases below 1 when collective reorientations increase, as measured by the decrease of the decay time of the autocorrelation of the cell velocity, τ_{dec} (Supplementary Figs. S9**d** and S10**d**). This deviation from Eq. 14 is weaker when hydrodynamics is included. Since we ensured that steady state is reached (in 1000 frames, 10 s of real time equivalent) before measuring the averages, we deduce that this discrepancy must come from detailed balance in the angular fluxes not being respected. Indeed, detailed balance assumes that:

$$P(s)\phi(s \to s + ds) = P(s + ds)\phi(s + ds \to s) \tag{16}$$

where $\phi(s \to s + ds)$ is the flux of cells changing their orientation from s to $s + ds$ over a small amount of time, and it is not necessarily satisfied when hydrodynamics- and collision-induced reorientations are concerned. Indeed, vortices are a net flux of orientation which thus cannot satisfy Eq. 16. Therefore, when hydrodynamics is included and even more so in the dry simulations, the effect of cell-cell interactions is not fully comparable to Brownian motion (for which detailed balance holds), and we prefer in this sense to talk about active enhancement of rotational diffusion, where $H\tau_\mathrm{E}\langle F - F_0 \rangle$ represents the drift if detailed balance was holding, and the ratio (15) is a measure of the effect of detailed balance breakdown.

Supplementary References

[1] Long, J., Zucker, S. & Emonet, T. Feedback between motion and sensation provides nonlinear boost in run-and-tumble navigation. *PLOS Comput. Biol.* **13**, e1005429 (2017).

[2] Wilson, L. G. *et al.* Differential dynamic microscopy of bacterial motility. *Phys. Rev. Lett.* **106**, 018101 (2011).

[3] Martinez, V. A. *et al.* Differential dynamic microscopy: A high-throughput method for characterizing the motility of microorganisms. *Biophys. J.* **103**, 1637–1647 (2012).

[4] Vladimirov, N., Lovdok, L., Lebiedz, D. & Sourjik, V. Dependence of bacterial chemotaxis on gradient shape and adaptation rate. *PLOS Comput. Biol.* **4**, e1000242 (2008).

[5] Yang, Y. L. & Sourjik, V. Opposite responses by different chemoreceptors set a tunable preference point in *Escherichia coli* pH taxis. *Mol. Microbiol.* **86**, 1482–1489 (2012).

[6] Dufour, Y. S., Fu, X., Hernandez-Nunez, L. & Emonet, T. Limits of feedback control in bacterial chemotaxis. *PLOS Comput. Biol.* **10**, e1003694 (2014).

[7] Celani, A., Shimizu, T. S. & Vergassola, M. Molecular and functional aspects of bacterial chemotaxis. *J. Stat. Phys.* **144**, 219–240 (2011).
